# Preceptors inspire students to be competent in nursing practice in a psychiatric public unit

**DOI:** 10.4102/hsag.v30i0.2899

**Published:** 2025-06-05

**Authors:** Bhekithemba Vellem, Karien Jooste

**Affiliations:** 1 Department of Nursing, Faculty of Medicine and Health Sciences, Walter Sisulu University, Mthatha, South Africa; 2 Faculty of Health Sciences, University of Limpopo, Polokwane, South Africa

**Keywords:** preceptors, supportive role, inspire, competent student nurses, psychiatric hospital, clinical practice

## Abstract

**Background:**

The preceptor’s role in nursing education remains important for its relevance in the professional development of student nurses and their advancement to becoming competent practitioners. Psychiatric nursing units address the management of complex mental health conditions that necessitate specific competencies from student nurses. It was unclear how the preceptors’ supportive role was experienced by student nurses placed in a psychiatric hospital.

**Aim:**

This study aimed to gain insight into the supportive role of preceptors in inspiring fourth-year student nurses to become competent in clinical practice at a South African public psychiatric hospital.

**Setting:**

The study was conducted at one of South Africa’s largest psychiatric public hospital, where fourth-year student nurses were placed to complete their psychiatric clinical nursing practice hours.

**Methods:**

The research employed a descriptive phenomenological design with individual interviews. Homogeneous purposive sampling was followed and 11 interviews with an interview guide were conducted at which stage data and meaning saturation were obtained. Interviews lasted between 30 min- 45 min and data analysis followed seven-step method.

**Results:**

Students experienced preceptors as unreplaceable in providing support during clinical practices. Professionalism, respect and inclusivity were the defining characteristics of the welcoming learning atmosphere that preceptors provided. Student nurses praised the preceptors’ willingness to respond to questions, treat them like professionals and support their required learning opportunities.

**Conclusion:**

In this study, participants reported a positive experience with preceptors, noting no challenges encountered.

**Contribution:**

This study contributes to the understanding of the critical supporting role of preceptors to student nurses in a psychiatric hospital.

## Introduction

In recent years, the field of nursing education has seen significant advances in the creation of new knowledge, technological advances and artificial intelligence (AI), aimed at the improvement of the quality of nursing care (Kavanagh 2021). Along with these changes, attention is increasingly more focussed on the role of preceptors in clinical training because of their influence on the professional development of student nurses to becoming capable nurse practitioners (Currie et al. 2019). Educational approaches currently aim to engage students in deeper thinking and teach them to use higher-order thinking and knowledge, compelled by competencies needed in the current clinical milieu (Forneris & Fey 2018). In clinical practices, effective preceptorship is an element of best clinical practices and a leading, supportive approach that is currently implemented in undergraduate nursing programmes worldwide (Dube & Rakhudu 2021; Lima & Alzyood 2024).

The term ‘preceptor’ has different connotations, and one of the meanings is being ‘a teacher, or instructor’ (Online Etymology Dictionary 2024). In nursing, preceptors are experienced practitioners, facilitating the integration of classroom learning into real-world practice (Shin, Park & Kim 2020). Preceptors act as facilitators for personalised *support* to undergraduate students in the clinical learning environment (Lima & Alzyood 2024). In this study, the role of the preceptors in supporting student nurses is emphasised, highlighting the importance of positive characteristics such as mindfulness and the ability to support students facing challenges in their workplace. This supportive role enables preceptors to help students maintain the energy necessary to perform effectively in their positions (Gardner 2023). Supportive characteristics in the role of a preceptor could include being positive, receptive and open to other’s ideas.

As the healthcare landscape evolves and becomes increasingly complex, the importance of effective preceptorship in preparing student nurses for the challenges of contemporary healthcare environments cannot be overstated (Dube & Rakhudu 2021). Despite the recognised significance of preceptorship globally, there remains a paucity of research that have explored the lived experiences of student nurses undergoing clinical training (Ashipala & Kampala 2022), particularly in specialised areas such as psychiatric nursing. Psychiatric nursing unlike other nursing specialities, presents unique challenges and opportunities for student nurses, requiring specialised competencies to effectively care for individuals with mental health disorders (Oremland, Louie & Napoles 2020). Understanding the experiences of student nurses engaged in psychiatric health clinical practices with preceptors is essential to gain in-depth insight into the supportive role that preceptorship plays in the professional growth and well-being of students in an ever-advancing nursing practice.

### Literature

Preceptorship is a cornerstone of nursing education, allowing students to apply theoretical concepts in real-world clinical practice settings, an inspiration for students to emerge as individuals. Preceptors must be competent to play an adequate supportive role that has different dimensions, which are highlighted in the ensuing discussion.

Kavanagh (2021) emphasises the ‘crisis in competency’ within nursing education, highlighting the necessity for effective preceptorship to ensure student nurses transition into competent practitioners. Preceptors are not merely supervisors but educators, mentors and role models who significantly influence and support students’ learning experiences and professional growth (Ashipala & Kampale 2022). It was found that a sense of belonging is fostered in students by preceptors who are approachable, respectful and professional (Rooke et al. 2022). It is critical for students’ learning process to feel at ease asking questions and seeking advice in an encouraging clinical practice setting (Bøe & Debesay 2021).

The ability of preceptors to tailor learning experiences to fit the specific requirements of student nurses is correlated with preceptorship effectiveness. Dube and Rakhudu (2021) reveal that students can recognise their areas of strength and growth with the assistance of preceptors who offer tailored comments, vital thinking and decision-making skills, through an individualised approach (Harrison et al. 2020). Studies have also revealed that students become more motivated and self-assured when preceptors treat them like unique individuals and show a sincere interest in their education (Ashipala & Kampale 2022; Kavanagh 2021). Preceptors bridge the gap between classroom learning and real-world practice by assisting students in applying theoretical concepts in a clinical setting through patient interactions and practical procedures (Dube & Rakhudu 2021; Tuvesson & Andersson 2021).

The student nurses’ professional identities are largely shaped by their interactions with their preceptors during these clinical practices. Positive experiences with preceptors can establish a sense of responsibility and commitment to patient care in students, encouraging them to embrace their duties as future nurses (Stănesu-Yadav & Lillekroken 2023). Another study reveals that students who experience a positive preceptorship relationship are more likely to form a solid professional identity and be passionate about nursing (Jassim, Carlson & Bengtsson 2022).

Despite the benefits of preceptors and their supportive roles during clinical practice, challenges exist in the preceptorship model. Nurse preceptors often face heavy workloads and may struggle to balance patient care with their educational responsibilities (Hong & Yoon 2021).

This can hinder their ability to provide the necessary support and guidance to student nurses. The preceptor’s role in inspiring student nurses to emerge as competent individuals in clinical practice is multifaceted and critical. Research indicates that while negative clinical experiences might increase anxiety and discourage students from continuing psychiatric nursing, positive experiences can create favourable attitudes about mental health nursing, enhancing self-confidence and interest in the field (Shaygan et al. 2023). As nursing education continues to evolve, the importance of preceptors in guiding and inspiring future nurses remains paramount (Hansen 2021). This research sought to shed light on; *‘What supportive role do preceptors play in inspiring fourth-year nursing students to become competent in clinical practice at a South African public psychiatric hospital’?*

This study aimed to gain insight into the supportive role of preceptors to inspire nursing students to become competent in clinical practice at a South African public psychiatric hospital.

The objective of the study was to:

- Describe the lived experiences of fourth-year students on the supportive role of preceptors in inspiring them to become competent in clinical practice at a South African psychiatric public hospital.

## Research methods and design

This study employed a descriptive phenomenological research design to investigate the supportive role of preceptors in the clinical practice of students placed in a psychiatric hospital. The choice of qualitative research was informed by its ability to delve deeply into participants’ lived experiences on the phenomenon (Creswell & Poth 2018) of the supportive role of the preceptor. The researcher followed the core principles of the descriptive phenomenological approach, which included bracketing, intuiting, analysing and describing the phenomenon (Alhazmi & Kaufmann 2022).

### Study setting

The research was conducted at a psychiatric public hospital in Cape Town Metropolitan Municipality, one of the largest of its kind in the southern hemisphere. It accommodated fourth-year undergraduate student nurses from a Western Cape higher education institution (HEI) who were accompanied by preceptors in their clinical component of the subject of Mental Health. Preceptors were nurse educators appointed by the hospital. Preceptors underwent a 3 months training programme in their role, which included the support to students in clinical teaching and learning during their clinical placement. They were each allocated to two to four students per month and conducted clinical assessments with students on a 1:1 basis in different psychiatric units.

### Population and sampling

The accessible population consisted of 42 fourth-year undergraduate student nurses from a HEI who were final year students at a psychiatric public hospital. Homogeneous purposive sampling was employed, which involved intentionally selecting participants of a site based on their similar characteristics (Crossman 2020). Participants were included based on:

- Having 3 months experience of partaking in a preceptorship programme in a psychiatric unit of the public hospital.
- Being a fourth-year student.

The sample size was established at the point that no new data emerged (data saturation) and the study sample was 11 participants.

### Data collection

Participants were recruited from the HEI where students were registered for their nursing programme. Ethical clearance was obtained from Ethics committee of a Faculty of Community and Health Sciences.

The researcher visited the School of Nursing at the HEI and explained the benefits and purpose of the study. Permission was asked for an information session with students, to explain the research study. A private room was also requested if students wished to be interviewed at the nursing school and had no other preference. Students received the contact details of the researcher and those who were interested in being part of the study could contact the researcher about their potential participation.

Before the main study, the researcher conducted two pilot interviews to assess whether the participants’ experiences would provide relevant insights into the research question (Keskitalo & Andersson 2023). No changes were made to the interview schedule and the data of the participants were included in the data analysis of the main study. Data collection was through conducting individual, unstructured interviews with an interview guide. The researcher posed a main question followed by probing questions based on the participants’ responses and used a digital recorder and wrote field notes.

The researcher obtained written informed consent from each participant before the interviews were held, which were scheduled in a private, quiet room to minimise distractions and ensure confidentiality. Their names were not mentioned on the recorder or field notes, ensuring anonymity. The researcher started with some informal questions to ensure that the participants felt comfortable and at ease to share their experiences with the researcher. The main research question was: *‘How was it for you to be supported by preceptors during your clinical practice at a psychiatric hospital?’*

Interviews lasting 30 min – 45 min provided sufficient time for participants to share their experiences and kept the interview focussed on the research question posed. The researcher probed by asking follow-up questions where applicable, thus gaining a deeper understanding of the participants’ experiences allocated to preceptors (Knott et al. 2022) in a psychiatric hospital setting. The researchers continued with data gathering until data and meaning saturation were achieved, ensuring that no new insights and meanings emerged from the data obtained during the interviews (Guest, Namey & Chen 2020).

### Data analysis

The researcher transcribed the recordings. The researcher employed Colaizzi’s (1978) seven-step process for qualitative data analysis (Polit & Beck 2021). Data triangulation of the transcripts, fieldnotes and observation was performed by the researcher for data analysis. The researcher initially read through all interview transcripts to gain a comprehensive understanding of the participants’ lived experiences, identified key statements and phrases, and formulated meanings into thematic clusters. The researcher consulted an independent coder to also do an analysis, after which a consensus meeting was held between the researcher and the independent coder. The researcher then developed an exhaustive description of the phenomenon, incorporating all emergent themes, and described the fundamental structure of the phenomenon to provide a clear depiction of the participants’ experiences.

### Trustworthiness

Trustworthiness pertains to confirmability, credibility, transferability and dependability. Credibility in this study was enhanced, as before the actual interviews, the researcher met with participants to explain the purpose, process and significance of the study. Member checking was conducted through transcribed data that needed to be validated with one participant after the interviews to ensure the accuracy of the information. The researchers bracketed their personal experiences to minimise potential biases (Woodberry et al. 2024). Regular consultation with the supervisor ensured adherence to the necessary research steps to ensure credibility of the results.

The supervisor conducted a rigorous process of in-depth checking to confirm the dependability of the findings. The themes were validated by referring to the original transcripts (Braun & Clarke 2023). The supervisor and the independent coder reviewed the transcripts, categories and theme formations. A consensus meeting was held between the independent coder (an expert on qualitative data analysis) and researchers. Confirmability was ensured by the field notes and recorded data. Transferability was made possible by the written detailed methodology in the report, and the thick descriptions of the results that were supported by participants’ quotes (Stenfors, Kajamaa & Bennett 2020).

### Ethical considerations

Ethical approval was obtained from the Higher Degrees Committee at a university in the Western Cape (Registration no:14/9/36). Permission was requested from the management of the selected institution to conduct the research study. The Research Ethics Committee of the selected psychiatric hospital granted permission for the study. Furthermore, permission was obtained from the Provincial Department of Health’s Research and Ethics Committee (Reference no: WC_2015RP44_840).

The participants were fully informed about the study, and the study was entirely voluntary, and participants could leave at any moment if they so desired. They were asked to sign an informed consent form.

The recorded data were stored on a compact disc, which will be kept for a period of 2 years after the research results have been published. The participants were given an information sheet, which spelled out the purpose and objectives of the study, benefits and any risks which would arise from participating in the study, and methods and processes of data collection and analysis. The digital recordings and field notes were transcribed and kept in the Cloud with a secure password. Only the researchers and independent coder had access to the data, and the files will be deleted from the Cloud after 5 years of completing the research. The final report used codes rather than names to protect participants’ identities, and only the data that were within the scope of the study were reported.

## Results

The study aimed to describe the preceptors’ supportive role in inspiring fourth-year nursing students to become competent in clinical practice at a South African psychiatric public hospital. The participants included both males (*n* = 3) and females (*n* = 8), all enrolled in undergraduate programmes and assigned to psychiatric units for clinical experience. These individuals were assigned to units with a preceptor assigned to them. The age range of the participants was 21–24 years.

### Theme 1: The supportive role of preceptors in inspiring students to emerge as individuals in clinical practice

In this study, the first theme identified was the supportive role of preceptors in inspiring students during clinical practice (Table 1). Preceptors’ approachability fostered team spirit and encouraged students to ask questions. By acknowledging students’ competencies, preceptors reinforced their readiness to enter the nursing profession. Their support enhanced students’ confidence to engage in diverse learning opportunities. In addition, students felt safe working with professional nurses who had completed formal preceptorship training programmes:

**TABLE 1:** Theme and categories obtained from the data.

Theme	Sub-themes
1. The supportive role of preceptors in inspiring students to emerge as individuals in clinical practice	1.1 The approachability of preceptors created a team spirit and an openness of students during the posing of questions 1.2 The respect of the preceptors in acknowledging the competencies of the student to enter the field as a professional nurse 1.3 The value of preceptors in enhancing student nurses’ confidence in participating in a variety of learning opportunities 1.4 Students felt safe work with registered nurses who had completed the formal preceptorship training programme

A participant felt supported in being able to reach her learning outcomes:

‘But I haven’t experienced any bad experience … feeling that they did not want me to achieve my objectives or that my input was not being taking into consideration. So no bad challenges, only good ones.’ (P5, female nursing student, 23 years)

This is consistent with the research conducted by Flott et al. (2022) regarding the actions of preceptors that promote a positive preceptorship experience.

#### Sub-theme 1.1: The approachability of preceptors created a team spirit and an openness of students during the posing of questions

Jassim et al. (2022) state that a team is crucial because it fosters a collaborative environment where learning responsibility is distributed among all members, including preceptors and student nurses, who promote shared knowledge and skills development. The importance of a two-way commitment was viewed as a collaborative team effort in creating student’s learning opportunities:

‘Ja, it is very good experience ja. It is kind of, it’s actually both and two sides. With staff side preceptors I mean and even the students as well, because if student we have to push hard to get to know what we have to know and the staff also have to try hard to help the student to know what they need, because at the end of the day you also becoming part of the team in the ward.’ (P3, female nursing student, 22 years)

An educational environment was created that made students felt at ease to ask questions:

‘I learned to be comfortable in a place, and the preceptor was good in that, mhm how can I say, he made the environment more comfortable so that you can feel comfortable to ask questions.’ (P4, male nursing student, 23 years)

Two participants highlighted that the preceptors allowed them to ask questions and explore new avenues that made them feel part of the team in the unit:

‘I was given the opportunity to ask questions, I was given the opportunity to go and find out, go and look at this, go and look at that, what is this, what is that? I was made part of it.’ (P5, female nursing student, 23 years)

‘And he has the experience in the ward rounds, and if there is something that we don’t understand in the ward rounds he come back to us to explain. Maybe if they are talking about those big words [*laughing*]. We will write those words down, and then he will explain to us.’ (P2, female nursing student, 23 years)

Preceptors allowed students the independence to practice their skills independently and they could consult and ask questions when they encountered any difficulties:

‘Mmh, sometimes the preceptor will supervise us, sometimes she won’t supervise us, for example, when we do activity group with the patients, we can do it alone, so she didn’t supervise … then if we have a question, we will ask her.’ (P9, female nursing student, 22 years)

These findings align with those of a study conducted in Ireland by Hardie et al. (2022), which similarly highlighted that student nurses may be perceived as more at ease and capable of learning as the preceptors’ positive attitudes, behaviour and support indicated that they wanted to nurture the students.

#### Sub-theme 1.2: The respect of the preceptors in acknowledging the competencies of the student to enter the field as a professional nurse

Hardie et al. (2022) found that building professional and respectful relationships during preceptorship is vital for effective clinical teaching and learning and together preceptors and students should be well prepared for an adult learning practice.

Respect during teaching and learning, between the preceptors and students, was two-ways:

‘Mmm it’s a very like professional relationship mm I respect a preceptor of what she does and also she, she respects me, due giving me, having opportunities but also if she sees like where I can improve, she will advise me on staff that I can do.’ (P10, female nursing student, 21 years)

The results revealed that students felt prepared to become professional nurses. The participants highlighted that they were acknowledged by the preceptors, who encouraged them to be involved in different activities and duties in the units:

‘Eeeh I had good, very good experience, they learn me a lot, eeeh I got involved, but they involved me with the stuff that I would like need to start doing next year [*community service year*].’ (P10, female nursing student, 21 years)

Students were entrusted with tasks that they thought they would only be encountered in after their final year of study, as community service nurses:

‘The staff they treat us, we are fourth-year students and what I like about the staff is they already treat us students as sisters already, so I like …’ (P6, female nursing student, 24 years)

‘Mmh she didn’t see us maybe as a threat, or some people to keep away from. She, she invited us to do many things with her.’ (P9, female nursing student, 22 years)

Participants reported that preceptors played a crucial role in their development, as they provided support that enabled students to use their knowledge, skills and attributes in the clinical context thereby facilitating the transition from novice to expertise practitioners:

‘Basically quite are quite easy approachable, they want to give you information, they want to show you how things are actually done here.’ (P1, male nursing student, 24 years)

‘I would say that a relationship between me and the operational manager, and two preceptors made me to be like act as sister myself in the ward, because they show us how to do things.’ (P8, female nursing student, 22 years)

Participants appreciated the welcoming atmosphere and being treated as adults in the various units, contributing to their positive preceptorship experiences. A participant uttered that from starting the first day in the unit, she was viewed as an adult:

‘Thank you very much, mhm … actually my experience in this ward in particular very, it’s very good so far from my first day that I started to work here, we were all welcome from our first day here and they really treated us as adults, as future professionals, so, so far, we really having a good relationship with our preceptors in the ward.’ (P3, female nursing student, 22 years)

#### Sub-theme 1.3: The value of preceptors in enhancing student nurses’ confidence in participating in a variety of learning opportunities

Girotto et al. (2019) found that nurse preceptors were committed to their role; they helped students develop critical thinking and autonomy while acknowledging the transformative potential of preceptorship. They see their main responsibility as educators who can improve students’ skills and attitudes by fusing theory and practice. The preceptors carefully planned and created learning opportunities for nursing students to learn. One participant said that preceptors skilfully organised instructional opportunities to introduce fresh concepts and activities, giving nursing students all-encompassing learning experiences:

‘Mhm, like, for example, last week we had to learn about seclusion, and the preceptor explained the whole procedure in detail to us and everything he organised for us to see the room for seclusion.’ (P4, male nursing student, 23 years)

The favourable encounters that student nurses had with their preceptors throughout their clinical placements indicated that ongoing essential assistance and direction were provided to them in their clinical learning situations. During their orientation at the psychiatry unit, the preceptors played a crucial role in instructing and mentoring the students on their unfamiliar surroundings:

‘I think it really contributes significantly to us, what we, they are really contributing because when we are here, we don’t exactly know everything that is going on in the ward, they orientate us well and guide us.’ (P3, female nursing student, 22 years)

According to Moroney et al. (2022), providing orientation to clinical assignments can have a substantial positive impact on students’ learning outcomes by promoting readiness, lowering anxiety and boosting motivation.

The broad scope of learning activities provided was mentioned by the participants:

‘The preceptor teaches us many things like many different kinds of activities and procedures related to psychiatry …’ (P9, female nursing student, 22 years)

Students were confident in how they performed their tasks independently or with direct guidance from the preceptors:

‘So what I like about these preceptors, is that they delegate and we do it. I like it because I know I have to do it, like for instance medication. If in the morning I must give medication I will come to medication, we as students we will come to medication because that is how they learned us.’ (P6, female nursing student, 24 years)

‘I am doing what is expected from me and there are no problems at all; when we have patient challenges, the preceptors guide and assist in handling them.’ (P6, female nursing student, 24 years)

Students were aware of the presence of the preceptor who allowed independent learning; however, they were always nearby when needed:

‘And discharging of patients to home now … so they actually show you how to do all of those procedures, so they are actually very much helpful.’ (P1, male nursing student, 24 years)

‘I did work in the intellectual disability ward so this was my first time to work in predischarge ward so new procedures that you need to do here, which is different from the ward I used to work in. So, they really are so kind and so helpful, they do show us how to admit the patients with criteria for admissions, they show us the procedures to be followed to discharge the patients to give them the leave of absence, things like that …’ (P3, female nursing student, 22 years)

#### Sub-theme 1.4: Students felt safe to work with registered nurses who had completed the formal preceptorship training programme

The results of this study showed that preceptorship training was beneficial for clinical nursing. One participant expressed gratitude for the preceptorship programme provided to registered nurses and suggested that it keep emphasising the development of a welcoming and secure learning environment for students:

‘I will say, like preceptorship, must continue to go on because, like sometimes you find it difficult to work with other registered nurses without it, they also need guidance on how to approach the students, so sisters who did the preceptorship, I find them more approachable and eeeh we learn a lot from them.’ (P9, female nursing student, 22 years)

‘Yes we gain a lot from the sisters who did the preceptorship.’ (P9, female nursing student, 22 years)

Hansen and Zuma (2024) highlight the importance of a successful clinical preceptorship in promoting students’ socialisation process and fostering the development of their critical thinking abilities:

A participant mentioned:

‘Ja preceptors are important, especially when it comes to orientation in psychiatry as most of us students are afraid of the psych, at practical, especially when they come for the first time.’ (P3, female nursing student, 22 years)

Despite being paired with a registered nurse who had completed a preceptorship, one participant reported not knowing that there were even trained preceptors. He did admit, although, that the preceptors were effective in addressing his learning goals. He said:

‘Mhm I was, I wasn’t aware that there were sisters here that actually went to the course for two weeks to actually to get knowledge and skills on how to deal with students, at UWC students, but they were fairly when, when we came here, they wanted to know what our objectives is.’ (P5, female nursing student, 23 years)

The importance of and need for trained preceptors came to the fore from the following statement:

‘Mhm If example … if more preceptors were to be included within this year or two-week preceptorship course … it will assist a lot for incoming students.’ (P5, female nursing student, 23 years)

## Discussion

The study’s findings emphasised how important preceptors were in providing student nurses with a supportive and stimulating learning environment while they were placed in clinical practice in a psychiatric hospital.

The approachability of preceptors fostered a team spirit that was mentioned to promote effective learning among student nurses in clinical settings. Literature adds that preceptors should be open to addressing students’ ongoing learning needs, for example, by answering their questions during troubleshooting sessions in a clinical setting (Girotto et al. 2019). Students felt welcome and free to pose questions and to engage in independent learning. Benny, Porter and Joseph (2023) state that preceptors who exhibit traits such as being available and giving feedback when needed, help students to feel at ease and capable of learning effectively when on clinical assignments.

Through the creation of an environment that was marked by professionalism, respect and inclusivity, preceptors fostered student engagement and helped them to feel respected as adults and essential members of the unit. Rooke et al. (2022), in their study, found that preceptors who create a respectful and supportive atmosphere allow students to participate in collaborative learning, which is crucial for their professional development. Students viewed preceptors as experienced, competent and committed to their role, which enhanced their self-confidence.

Preceptors helped students develop a sense of respect and preparedness for professional practice by involving them in various clinical tasks usually assigned to seasoned practitioners (Tuvesson & Andersson 2021). The findings indicated that students were treated as adults and allocated tasks already completed by a professional registered nurse. Ebu Enyan, Boso and Amoo (2021) found that preceptors should fulfil their crucial role in the clinical training of nursing students by respecting them as adults and new entering nursing professionals, which motivates them and enhances their learning experience.

The participants in this study were exposed to numerous learning opportunities throughout their accompaniment with preceptors at a public psychiatric hospital. The participants expressed gratitude for their preceptors’ support and direction, which made their learning process uncomplicated. Rosli, Choo and Idris (2022) discovered that students’ confidence increases when they feel supported by their preceptors. A supportive and encouraging environment fosters critical thinking and skill development in addition to assisting students in overcoming clinical obstacles. Students valued the qualities of the preceptors in assisting them to reach their learning outcomes. According to a study performed by Stănesu-Yadav and Lillekroken (2023), preceptors enhancing clinical learning opportunities increase students’ clinical competence and build their confidence as they are better equipped to manage patient care scenarios.

Preceptors are invaluable in helping students apply their knowledge in real-world contexts. Benny, Porter and Joseph (2024) suggest that structured support and guidance provided by trained nurse preceptors significantly enhance the learning experiences of nursing students, thereby making their clinical placements more effective. Preceptor-trained registered nurses are found to be more capable of establishing a supportive learning atmosphere and meaningful learning experiences for students. Preceptors who receive this training are more equipped to support students during their clinical education (Vuckovic, Carlson & Sunnqvist 2021). The findings indicated the need for students to understand what preceptorships mean. According to Tuvesson and Andersson (2021), preceptors are responsible for clarifying their roles to student nurses. This includes providing instructions, demonstrating tasks and promoting learning activities that support the goals of the pupils.

The results highlight the supportive role that highly qualified preceptors play in providing student nurses with excellent clinical experiences, especially in specialised settings such as psychiatric hospital. Preceptors are essential to student nurses’ clinical education, especially in demanding fields such as psychiatry. A lot of students worry about their first encounters in psychiatric settings because they are afraid of the intensity of the setting and the complexity of patient interactions. The study promotes continued investment in preceptorship training to guarantee that every student nurse has encouraging and stimulating learning opportunities (Tuvesson & Andersson 2021). This investment can improve the overall quality of treatment in hospital settings and improve students’ educational experiences during clinical placement at a psychiatric hospital.

### Recommendation

This study recommends that a standardised 1-week preceptorship training programme is specifically developed for psychiatric public training sites by HEI with input from area managers in public hospitals. The programme will be attended by professional nurses entering a preceptor role. Furthermore, it is recommended that HEIs inform preceptors and hospital nursing management about any modifications to the curriculum or student objectives. This will allow them to make necessary adjustments to their practices. Refreshing updated programmes can integrate changes in the curriculum along with a feedback from preceptors, ward staff and students. This should provide 4th year students with the opportunity to be taught within a similar theoretical framework, and preceptors with the knowledge and skills necessary to support student nurses effectively during their clinical placements. It is important for psychiatric hospitals and HEIs to have a cooperative team environment that fosters a helpful and encouraging environment for the teaching and learning of student nurses.

### Further research

A study can performed on the impact played by preceptors in making final year student nurses to be ready for their community service year.

### Study limitations

The limitations of the study were identified as the small sample, only one hospital was included and only one HEI was used. Although the study’s findings are rich, they cannot be generalised to other settings.

## Conclusion

This study sought to investigate the supportive role that preceptors played in being a support to student nurses placed in clinical practice at a South African psychiatric hospital. The study’s conclusions show that undergraduate nursing students experienced a well-designed preceptorship programme including good relationships with preceptors at a psychiatric hospital. Preceptors were viewed as crucial in establishing a professional, courteous and inclusive learning atmosphere that encouraged student participation and made them feel like vital members of the medical team.
